# Split-root systems: detailed methodology, alternative applications, and implications at leaf proteome level

**DOI:** 10.1186/s13007-020-00706-1

**Published:** 2021-01-09

**Authors:** Iñigo Saiz-Fernández, Martin Černý, Jan Skalák, Břetislav Brzobohatý

**Affiliations:** 1 Phytophthora Research Centre, Department of Molecular Biology and Radiobiology, Faculty of AgriSciences, Mendel University in Brno, Zemědělská 3, 613 00 Brno, Czech Republic; 2 Department of Molecular Biology and Radiobiology, Faculty of AgriSciences, Mendel University in Brno, Zemědělská 3, 613 00 Brno, Czech Republic; 3 CEITEC–Central European Institute of Technology, Mendel University in Brno, Zemědělská 1, CZ-61300 Brno, Czech Republic; 4 Institute of Biophysics of the Czech Academy of Sciences, Královopolská 135, 61265 Brno, Czech Republic; 5 Present Address: Functional Genomics & Proteomics of Plants, CEITEC MU, Central European Institute of Technology, Kamenice 5, 625 00 Brno, Czech Republic

**Keywords:** *Arabidopsis thaliana*, Drought stress, Proteomics, Phytohormones, Split-root systems

## Abstract

**Background:**

Split-root systems (SRS) have many applications in plant sciences, but their implementation, depending on the experimental design, can be difficult and time-consuming. Additionally, the system is not exempt from limitations, since the time required for the establishment of the SRS imposes a limit to how early in plant development experiments can be performed. Here, we optimized and explained in detail a method for establishing a SRS in young *Arabidopsis thaliana* seedlings, both in vitro and in soil.

**Results:**

We found that the partial de-rooting minimized the recovery time compared to total de-rooting, thus allowing the establishment of the split-root system in younger plants. Analysis of changes in the Arabidopsis leaf proteome following the de-rooting procedure highlighted the distinct metabolic alterations that totally and partially de-rooted plants undergo during the healing process. This system was also validated for its use in drought experiments, as it offers a way to apply water-soluble compounds to plants subjected to drought stress. By growing plants in a split-root system with both halves being water-deprived, it is possible to apply the required compound to one half of the root system, which can be cut from the main plant once the compound has been absorbed, thus minimizing rehydration and maintaining drought conditions.

**Conclusions:**

Partial de-rooting is the suggested method for obtaining split-root systems in small plants like *Arabidopsis thaliana*, as growth parameters, survival rate, and proteomic analysis suggest that is a less stressful procedure than total de-rooting, leading to a final rosette area much closer to that of uncut plants. Additionally, we provide evidence that split root-systems can be used in drought experiments where water-soluble compounds are applied with minimal effects of rehydration.

## Background

A split-root system (SRS) is formed by a plant whose root has been split into different compartments, which are isolated from each other [1]. The great advantage of this arrangement is that it allows the differential treatment of separate parts of the root system while sharing a common aerial part. Thus, SRSs provide a way to simulate the heterogeneity, both horizontal and vertical, inherent to field conditions. This technique has been successfully employed for the study of several aspects of plant metabolism and development since as early as the 1940 s [2], one of the most iconic being the discrimination of systemic versus local regulation mechanisms [1].

Depending on the plant species being studied and the objective of the study, there are different strategies to establish the SRS. In the case of studies involving vertical soil heterogenicity, a single root system can be arranged to grow through the different compartments of the system [3, 4]. In order to simulate horizontal soil heterogeneity, on the contrary, it is necessary to create two or more physically separate root systems [1]. In plants with more than one seminal root, like wheat (*Triticum aestivum*), these roots can easily be diverted into different compartments right after germination [5]. However, in those species with only one primary root, such as *Arabidopsis thaliana*, maize (*Zea mays*), pea (*Pisum sativum*) or *Medicago truncatula* [6–8], the process is usually not that straightforward. For experiments requiring plants in the later stages of development, it is sometimes possible to divide the fully developed root system into the different compartments, without any prior procedure [9]. However, for studies performed on young plants this approach may not be suitable. In these situations, generally, seeds are germinated and plantlets are first grown until they develop a main root. Subsequently, the root is longitudinally cut and the two root sections are then grown in independent pots until they fully develop [6]. Alternatively, the main root can be removed to induce secondary roots that will then be split in two separate environments [8]; or it can be cut right below the two first lateral roots, which will later be separated into different compartments [10]. One even more advanced method, referred as inverted Y-grafting, involves making a small incision on the hypocotyl of a plant and inserting on it the excised roots of another [8, 11], or performing oblique cuts on the hypocotyls of two plants and binding the rootstocks together in a V-shape slit into which the excised apex of a third plant is inserted [12], thus yielding a plant with two root systems. However, this method is very skill-demanding and grafted plants display low survivability rates [11].

When thinking about SRS, perhaps the first image that comes to the mind is that of two pots stacked together, with the plant growing on the edge between them, as shown by Marino et al. [6], Kassaw and Frugoli [8], and many others. However, this is not the only possible arrangement. Depending on the nature of the experiments, a broad range of compartmentalization techniques to separate the split-roots in various growth systems has been used [8]. For instance, one single pot vertically divided with a plastic partition has been employed in wheat [5]. PVC (polyvinylchloride) piping elbows have been used in soybean (*Glycine max*) and split root tubes have been used in soybean and vetch (*Vicia sativa*) [8], while PVC vessels containing a polyethylene bottle in the centre have been successfully employed in *Ricinus communis* [13]. Lastly, split root agar plate assays were performed using *Trifolium subterraneum, Lotus japonicus* or *Arabidopsis thaliana* by separating the roots with plastic dividers [10] or by removing the centre of the agar to create separate root environments [14]. It is also worth mentioning the clever use of “net pots” shown by Dodd [11], which allows the development of the split roots into a plastic container that can be easily transferred into the required heterogeneous growth conditions with minimum plant disturbance.

The existence of such varied methodologies highlights the plasticity inherent to SRS, which allows it to be integrated into larger studies, where it can serve as a complementary tool. However, its implementation is not free of complications and it can be very time consuming, particularly for those new to it. This work tries to provide information to help researchers during their initial work with SRS. In addition, we will show potential and “unorthodox” applications of SRS other than those commonly described.

Lastly, one aspect of working with SRS that should be considered is the effect that the procedures leading to the establishment of such arrangements may have on plant performance during the SRS set-up and/or subsequent treatment. Given the extreme adaptability of plant growth to most of the external conditions, plants are able to adapt to the SRS and resume normal growth [1, 8]. However, when analyzing the result obtained using SRS it is important to take into account that, as a side-effect of the undergone procedures, plants may be more or less tolerant to certain treatments such as drought, salinity or pathogen attack, in a similar way to that observed in grafted plants [15, 16]. Moreover, considering that complete de-rooting, as opposed to plant decapitation, is not common stress in nature and when it happens it more often than not leads to plant death, this kind of stress has rarely been studied. Therefore, this study offers insight into how partial and complete de-rooting procedures modify the Arabidopsis leaf proteome, both at the time of the cutting and during the subsequent healing process. This information will thus be of interest to researchers, not only during the planning and execution of experiments involving SRS, but also during the interpretation of the data obtained from such experiments.

## Results and discussion

The first step to establish an SRS is to choose a method that would be appropriate for both the species under study and the purpose of the experiment. The objective of the present study was to establish an SRS in Arabidopsis plants when they are as young as possible. Previous literature suggested five possible methods, each one displaying different advantages and disadvantages, as summarized in Table 1. Unfortunately, most are inadequate for the requirements of the present study, except for the method of cutting the whole root of the young seedlings and splitting the newly forming lateral roots, as described by Kassaw and Frugoli [8]. However, as the early establishment of the SRS was one of our main goals, some modifications were implemented into the method. Most notably, the previous procedure of cutting the roots at the shoot-to-root junction (totally de-rooted plants-TDR) was compared with a similar procedure in which the cut was done approximately half centimetre below the said junction, thus leaving a part of the main root attached to the shoot (partially de-rooted plants-PDR).

**Table 1:** Summary of the advantages and disadvantages of different methods for the establishment of a split-root system available in the literature

Method	Source	Destructive procedure required?	Technically challenging?	Achievable in young seedlings?	Applicable in Arabidopsis?	Schematic representation of the technique
Splitting of the developed root system	[9]	No	No	No	Yes	
Splitting of newly forming lateral roots	[5]	No	No	Yes	No	
Cutting longitudinally and Splitting the main root	[6]	Yes	Yes	Yes	No*	
Inverted Y-grafting	[11]	Yes**	Yes	Yes	Yes	
Cutting the whole root, splitting the new roots	[8]	Yes	No	Yes	Yes	

* Unless the researcher possesses surgeon-like skills

** Even though no part of the plant is removed, it requires making an incision on the root in order to insert the graft

### The type and time of the cut greatly affect further plant development

**Fig. 1:**
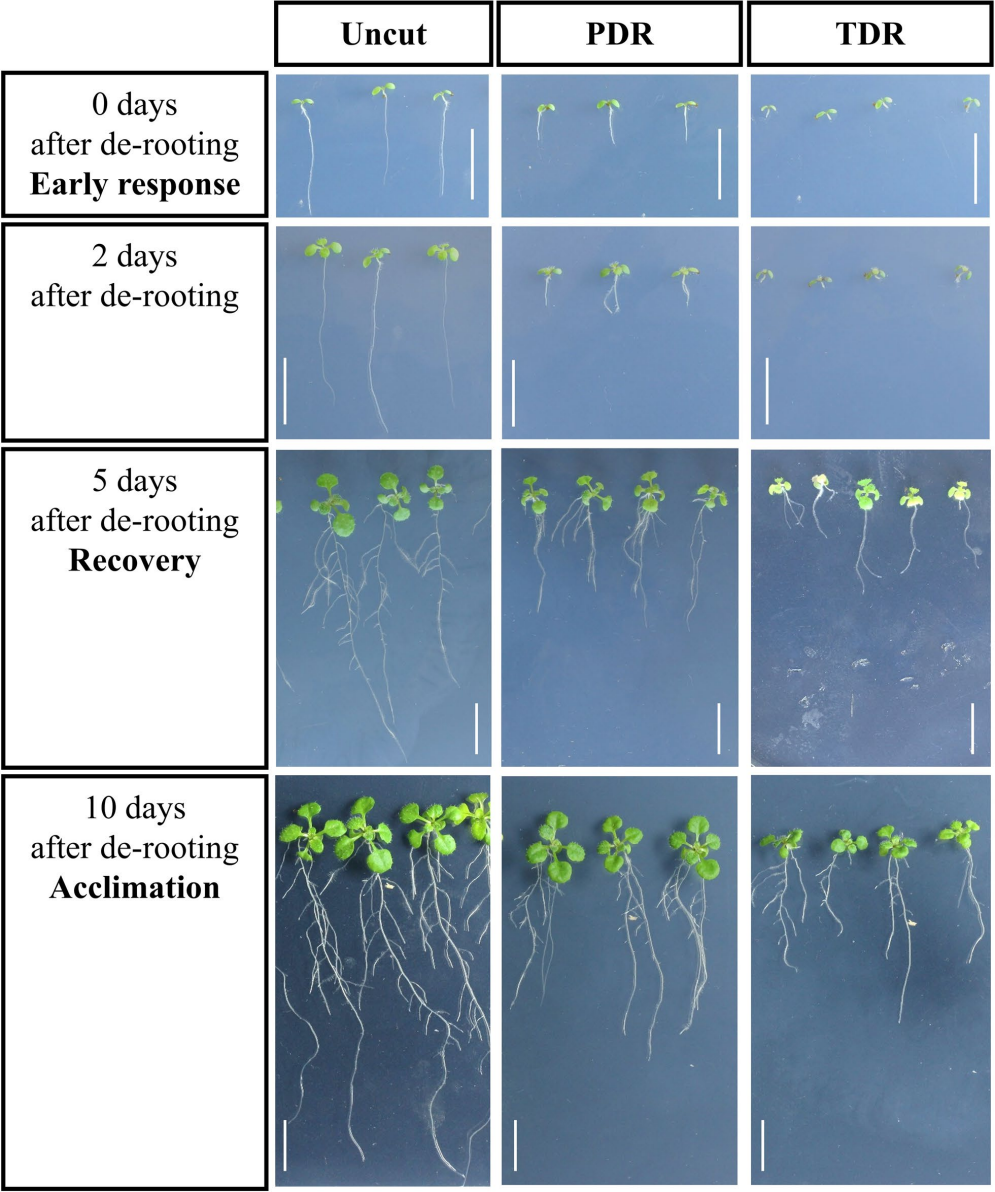
Visual comparison of the effect of total de-rooting (TDR) versus partial de-rooting (PDR), performed 7 days after sowing, on the development of new roots in *Arabidopsis thaliana* plants. The white bar is equivalent to 1 cm

**Fig. 2:**
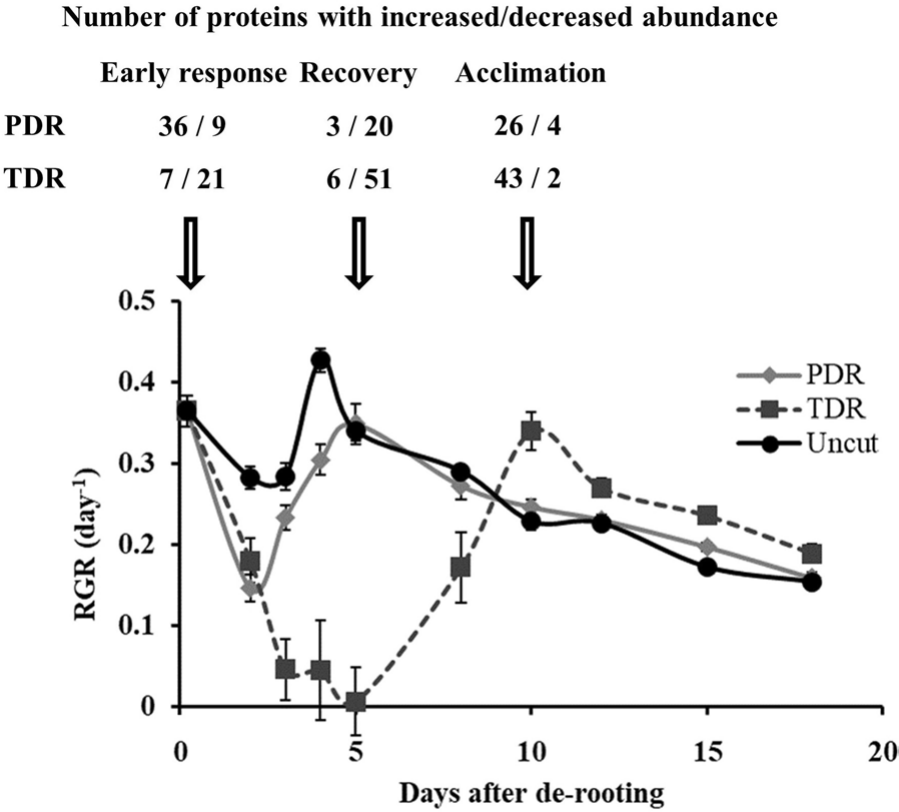
Effect of partial (PDR) and total de-rooting (TDR), performed 7 days after sowing, on rosette relative growth rate (RGR) and on the number of leaf proteins with increased/decreased abundance (compared to uncut plants) during early response (4 h after de-rooting), recovery (5 days after de-rooting), and acclimation (10 days after de-rooting). Arrows indicate the time at which the samples corresponding to each of these three phases were collected

The procedure of partially or completely de-rooting Arabidopsis plants had a profound effect on the subsequent plant development (Figs. 1 and 2). The recovery time, defined as the time past between plant de-rooting and the regain of relative growth rates equal to uncut plants, was significantly shorter in PDR plants than in TDR plants, leading to higher leaf area (Table 2) and more developed root system (Fig. 1). Therefore, the newly formed roots of PDR plants were long enough for their transfer into the SRS earlier than those of TDR plants. Additionally, the survival rate was much higher in PDR plants, which not only increases the chances of finding plants with rootstocks adequate for SRS establishment, but is also indicative of the lower stress that partial de-rooting imposes on plants, compared to total de-rooting.

**Table 2:** Effect of TDR and PDR on final rosette area, recovery time, and plant survival rate

	Final area (mm^2^)	Recovery time (days)	Survival rate (%)
TDR			
Time of the cut			
4 DAS	145 ± 12 b	8.5 ± 0.3 b	88 ± 5 a
6 DAS	132 ± 14 bc	7.5 ± 0.4 c	73 ± 8 abc
7 DAS	136 ± 12 b	7.4 ± 0.3 c	77 ± 5 a
9 DAS	109 ± 10 cd	7.6 ± 0.3 c	59 ± 5 bc
10 DAS	117 ± 14 bc	8.8 ± 0.5 b	51 ± 7 c
11 DAS	77 ± 11 de	10.7 ± 0.6 a	62 ± 8 bc
15 DAS	67 ± 8 e	12.1 ± 0.5 a	77 ± 8 ab
Uncut	290 ± 7 a	n.a.	100 ± 0 a
PDR			
Time of the cut			
4 DAS	209 ± 13 bcd	6.5 ± 0.4 a	95 ± 3 ab
6 DAS	227 ± 9 bc	5.2 ± 0.2 c	93 ± 4 ab
7 DAS	228 ± 12 bc	6.2 ± 0.2 a	100 ± 0 a
9 DAS	220 ± 7 bc	6.1 ± 0.3 ab	97 ± 3 ab
10 DAS	230 ± 10 b	5.7 ± 0.4 abc	97 ± 3 ab
11 DAS	195 ± 11 cd	5.3 ± 0.2 bc	87 ± 6 b
15 DAS	193 ± 6 d	5.9 ± 0.5 abc	91 ± 6 ab
Uncut	290 ± 7 a	n.a.	100 ± 0 a

Different letters mean statistically significant differences (within each cut type) for p < 0.05, according to Student’s *t* test (n ≥ 10)

DAS: days after sowing; TDR: Total de-rooting; PDR: Partial de-rooting; n.a.: not applicable

Final leaf area (mm^2^) was estimated 24 days after sowing. Recovery time is the number of days required for the plants to get their relative growth rate equal to uncut plants

The time after sowing at which the de-rooting procedure was performed had a drastically different effect on TDR and PDR plants (Table 2). In the case of TDR plants, delaying the time of de-rooting sharply decreased final leaf area, particularly if it was performed past 10 DAS. When the procedure was performed 11 or 15 DAS, TDR plants presented extremely decreased rosette areas, together with significantly extended recovery time. Surprisingly, the survival rate did not entirely follow the same pattern, as plants cut 15 DAS did not have significantly lower survival rate than those cut early in plant development. Instead, the lowest survival rates were found in TDR plants that had been cut between 9 and 11 DAS. The reason for this decrease in survival rate seems to be the plant developmental stage, since these plants, which were at the four-leaf-stage, had all leaves of a very similar size. Consequently, it was very difficult to make the end of the hypocotyl to stay in contact with the growth medium following the de-rooting procedure. Without being able to take water and nutrients from the medium, most of these plants either dried to death or fell to the bottom of the petri dish. In contrast, the time of de-rooting did not have such a dramatic effect in the case of PDR plants. The recovery time and survival rate were fairly similar among all PDR plants, while the final rosette area was decreased only in PDR plants cut 11 and 15 DAS (Table 2).

### Total and partial de-rooting trigger distinct proteomic responses

**Fig. 3:**
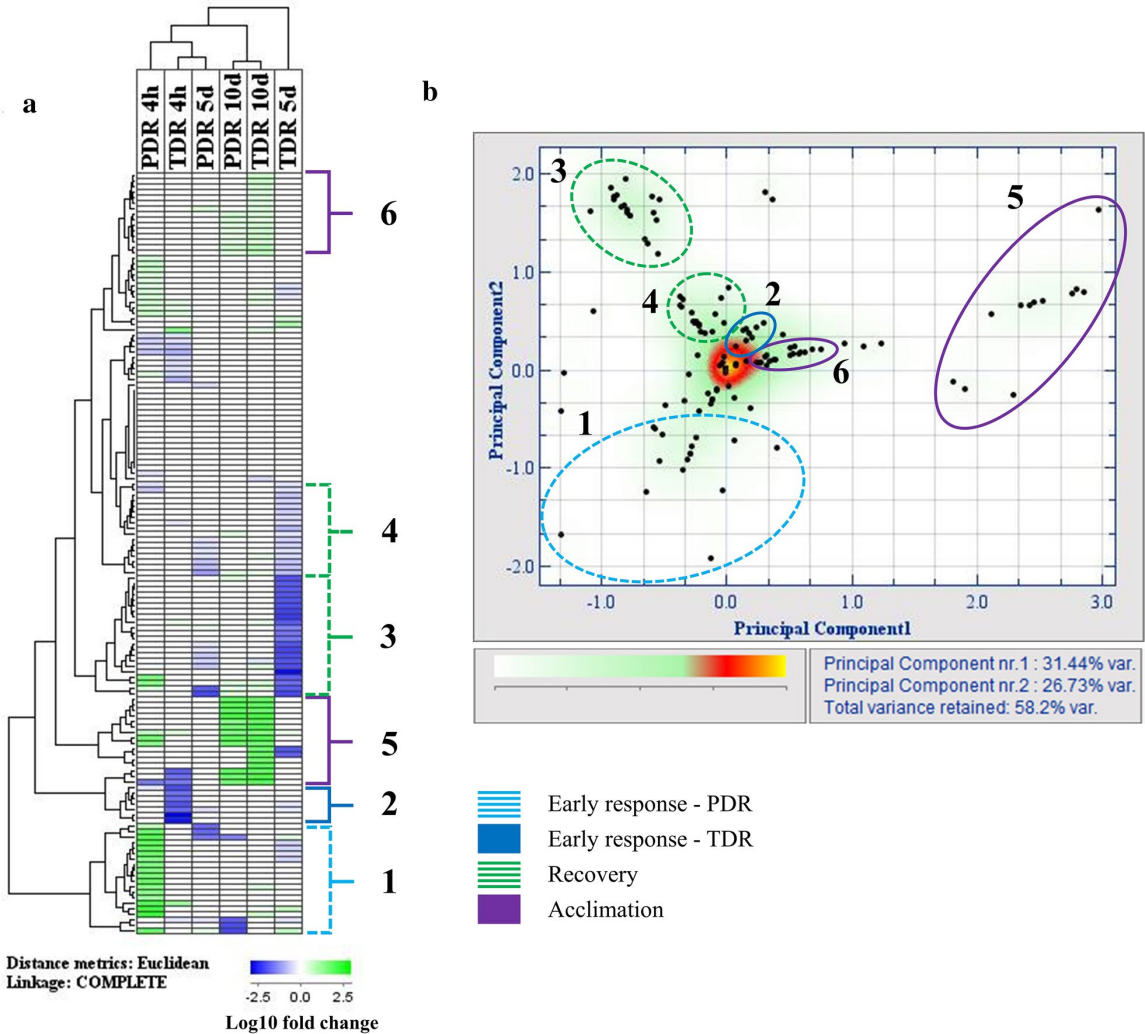
Heatmap (**a**) and principal component analysis (PCA, **b**) of the variations in the abundance of leaf proteins in partially (PDR) and totally de-rooted (TDR) plants, compared to uncut plants, 4 h, 5 days, and 10 days after de-rooting. The brackets and corresponding circles shown in **a** and **b**, respectively, highlight the clusters of proteins that were affected at the different stages following plant de-rooting

Despite the many advantages that SRSs offer to plant sciences, it is important to take into account the impact they may have in subsequent plant development; particularly in those methods in which plants need to be subjected to any kind of destructive procedure prior to the establishment of the SRS (Table 1). Here, we describe the leaf proteomic alterations that Arabidopsis plants undergo following the partial or total de-rooting required for the establishment of an SRS. This data revealed differences in the protein regulation, not only between TDR and PDR plants but also between the different stages of the stress response: early response, recovery and acclimation (Fig. 2, Additional file 1: Tables S1–S4). Briefly, these three stages correspond to 4 h after de-rooting, the time at which the relative growth rate (RGR) of TDR plants started to recover (5 days after de-rooting), and the time at which the RGR of TDR plants equalled uncut plants (10 days after de-rooting), respectively. Each one of these stages was characterised by the regulation of different proteins, which can be seen clustered together in the heatmap and PCA shown in Fig. 3.

#### Early response

The first few hours after the imposition of an external stress are commonly considered as the early response phase [17], and are crucial for plant survival [18, 19]. Interestingly, plants subjected to partial and total de-rooting behave rather differently regarding the changes undergone by their respective leaf proteomes. While in PDR plants increase in protein abundance is predominant, in TDR plants the early response seems to be driven mostly by a decrease in protein abundance (Fig. 2). This difference in the early response is highlighted by the separate clustering of the proteins affected at this stage in PDR and TDR plants shown in Fig. 3b. However, despite the differences in regulation between PDR and TDR plants, the main changes observed in Arabidopsis leaf proteome during those few hours after de-rooting can still be grouped into one of the following categories: stress response and adjustment of plant metabolism.

The stress response observed during the early response phase highlights the increased abundance of proteins related to stresses that involve mechanical damage (Additional file 1: Table S1). These proteins take part in a wide range of metabolic pathways, but all of them have been described to mediate the response against wounding derived from either pathogen, insect or herbivore attack [20]. At a tissue level, some effects of wounding are very similar no matter what the source of the stress is (i.e. cell damage, membrane breakage, mixing of previously compartmentalized molecules, disconnection between plant conductive tissues) [21–23]. Therefore, we can find similar response mechanisms against all of them [24, 25], including de-rooting.

However, even if the local effect of the wounding is similar, the implications at plant level are very different. When a plant suffers the attack of above-ground insects or herbivores it usually faces damage to the aerial tissues (leaves and stems), which results in a reduced photosynthetic area that needs to be restored, but the root system is left mostly intact [23, 26]. On the contrary, de-rooted plants suffer no immediate damage to their photosynthetic tissues but are left with a severely reduced root area (non-existent in the case of TDR plants), which will greatly compromise their water and nutrient uptake capabilities [27]. This is very similar to the effect observed in plants that have been attacked by root herbivory [26]. The consequences of such damage to water uptake and transport structures can persist for weeks after the initial injury and lead to leaf desiccation [28]. Therefore, an increase in proteins common to most abiotic stresses can be observed, including those involved in water deprivation, mostly HSPs (Additional file 1: Table S1), which are responsible for maintaining protein stability and membrane integrity [29]. Even though HSPs were originally thought to only act during heat stress, their involvement in other stress responses, particularly wounding, was later proved [30]. Interestingly, Cheong et al. [30] showed how the transcription of genes encoding HSPs during wound stress followed a similar time frame to that of the normal heat stress response. It is worth mentioning that, despite increased abundances in both PDR and TDR stress protein groups, increases were greater in PDR plants. This result is in line with the findings of Mody et al. [31], which suggest that plant response may not increase linearly with the magnitude of the stress and that, after some threshold is reached, different defence mechanisms may be triggered.

When plants are subjected to water shortage, one of the first reactions is the stomatal closure, which limits water loss but also decreases gas exchange, leading to a decrease in CO_2_ assimilation [32]. Thus, plant wounding can lead to the uncoupling between photosynthetic electron transport and carbon assimilation [33], and therefore to the production of reactive oxygen species (ROS) [34]. This was particularly relevant for TDR plants, since they suffered a complete loss of water-absorbing surface and would need more time until adequate water supply was restored. Therefore, in order to minimize the damage caused by ROS, de-rooted plants needed to make major adjustments to their carbon metabolism, as it has also been described by Nabity et al. [22]. During the early response phase TDR plants showed a decrease in proteins related to chlorophyll synthesis and subunits of the photosystem I (Additional file 1: Table S2), probably as an attempt to adjust light absorption to the decreased gas exchange and carbon assimilation rates [35] and possibly reduce ROS production [36]. It was also observed a decrease in mitochondrial and chloroplastic ATP synthase subunits (Additional file 1: Table S2). Since electron transport chains are structures prone to ROS production during biotic and abiotic stresses [37, 38], this reduction in ATP synthases could be either a consequence of damage from ROS, as suggested by Yan et al. [39], or a down-regulation intended to limit the production of such compounds.

Contrarily, the water deprivation suffered by PDR plants was not as severe as in TDR plants. Since PDR plants kept part of their root system, some water uptake was still possible [40] and, additionally, the recovery time was significantly shortened (Table 2). As a consequence, PDR plants did not undergo the same repression of light-harvesting and energy-producing pathways (Additional file 1: Table S2). In these plants, there was an up-regulation of the amino acid metabolism, tricarboxylic acid cycle, and protein catabolism (Additional file 1: Table S3), which is a response previously found in water-deprived Arabidopsis plants [41]. In addition, there was a further increase in proteins related to the remobilization of carbon from starch. The decrease in starch content is a known response of plants against water deprivation [42]; but in the case of de-rooted plants it could also serve as a way to relocate carbon compounds from the aerial part to the roots and restore root growth [43], since wounding is known to strongly affect plant source-sink relationship [26, 36, 44]. In fact, Correa et al. [45] have already highlighted the importance of soluble sugar on the restoration of root growth, and Robert et al. [46] have shown that maize plants allocate photoassimilates from leaves to the stem as a prelude to root regrowth. In addition, the catabolism of starch and other energy storage compounds to provide carbon and energy for the production of defense-related metabolites is a common response upon herbivore attack [47].

#### Recovery

The recovery phase corresponds to the point at which plants had regained a functional root system (Fig. 1), and their relative growth was either equivalent to uncut plants (PDR plants) or trending towards that point (TDR plants) (Fig. 2). While the early response phase was quite different between PDR and TDR plants, the recovery phase was more comparable and was driven mostly by decreases in protein abundance (Figs. 2 and 3a). In fact, there was a decrease in proteins related to phenylpropanoid and glucosinolate metabolism (Additional file 1: Table S1-AT3G14210.1, AT3G24503.1, AT4G37980.1, AT5G42100.1); metabolic pathways that are known to play crucial roles in plant stress response, particularly those involving tissue damage [48, 49]. This decrease could be an indicator of plants having overcome the worst part of the de-rooting stress. The return to control levels of most of the stress-related proteins discussed in earlier (Additional file 1: Table S1) supports this hypothesis.

However, the recovery of photosynthesis after water deprivation can be a slow process, taking from days to weeks [50]. In fact, leaf proteomic analysis suggests a stronger down-regulation of carbon assimilation in the recovery phase compared to the early response phase, both in TDR and, particularly, in PDR plants (Additional file 1: Table S2). Additionally, Johnson et al. [26] have pointed out that root damage can trigger an increase in the translocation of photoassimilates towards the re-growing tissues. As a consequence of these two effects, there was a marked decrease in the concentration of most forms of soluble sugars in both treatments (data not shown).

It is interesting to note that most of the proteins whose abundance decreased during the recovery were different from those altered during the early response (Fig. 3a). This suggests that Arabidopsis plants deploy different response mechanisms at various stages of post-derooting healing. Most likely, the early response is governed mainly by the signals derived from the mechanical damaged inflicted while de-rooting and focuses on preparing plant metabolism for any possible infection [51] and for the re-building of the lost tissues [52]. While it also includes adjustments to carbon metabolism and light-harvesting structures in prevision of water shortage and photooxidative damage, this part of the early response is mostly of a preventive nature, since plants had not actually gone through any water deprivation yet [53, 54]. On the contrary, when the plants reached the recovery phase they had already suffered the side effects associated with de-rooting (water shortage, stomatal closure, decrease in gas exchange, etc.) and had needed to make long term adjustments in carbon metabolism.

#### Acclimation

The acclimation phase corresponds to the period in which all the treatments had regained relative growth rates comparable to those from uncut plants (Fig. 2). As opposed to the decrease in protein abundance that predominated during the recovery phase, the acclimation phase is highlighted by the increase in protein abundance all over the different metabolic pathways, with almost no decreased proteins compared to the uncut, control plants (Figs. 2 and 3a). Particularly, it was observed an overall increase in proteins related to photosynthesis, both in PDR and TDR plants (Additional file 1: Table S2), suggesting that at this stage plants had finally overcome the effects of the stress generated by the de-rooting. This idea is supported by the increase in the concentration of LL-diaminopimelate aminotransferase (Additional file 1: Table S1), a protein linked to systemic acquired resistance [55]. According to Song et al. [56], the increase in LL-diaminopimelate aminotransferase promotes Arabidopsis development and suppresses its defences once the stress has been overcome. Similarly, the increase observed in ALDH1a, an enzyme taking part in the phenylpropanoid metabolism, could indicate increased salicylic acid production, which is itself responsible for the plant systemic acquired resistance [57]. In addition, there was also a marked increase in proteins related to the tricarboxylic acid cycle and glycolysis (Additional file 1: Table S3), suggesting an increase in the in situ use of carbon resources once that the root system had been restored and required a smaller import of assimilates [58]. All these changes in leaf proteome suggest that plants had effectively recovered from the stress inflicted by the de-rooting procedure.

#### Proteins show a high healing phase-specificity

Plant healing after de-rooting is a complex process, which involves the fine-tuning of most metabolic pathways at every step in order to assure plant survival. To this regard, there appears to be a high specificity between each of the affected proteins and the various stages of post-derooting development, with little overlap between stages (Fig. 3a). Therefore, the proteins responsible for plant response at each stage of healing show quite a high level of clustering in the principal component analysis (Fig. 3b). This is coherent with the observations made by Cheong et al. [30], which showed a mere 10% of overlap in the gene transcription during distinct phases of wound stress response. Interestingly, despite the high healing phase-specificity, the overall leaf proteomic profile was not that different between TDR and PDR plants. Both treatments showed similar profile in the key proteins regulated during recovery and acclimation (Fig. 3a, clusters 3 to 6), despite the number of affected proteins being higher in TDR plants. The exception to this trend is the early response, during which PDR and TDR plants showcase a distinct response, as shown by the separate clustering from each other (Fig. 3b).

It is worth noting that the roots initially originated after the de-rooting procedure were different in TDR and PDR plants. In the case of PDR plants, the new root system started from lateral roots, originated from the remaining part of the root, while in TDR plants the first roots to appear were adventitious roots, originated directly from the hypocotyl. Even though both types of roots can support normal shoot development [8, 10] and eventually evolve into mature root systems that are functionally indistinguishable from each other [59, 60], during the early stages of development these two types of roots can have different absorption rates for different nutrients [61]. This is something that should be taken into account in experiments performed a short time after the appearance of the new roots, even though the use of proper control plants can minimize the introduction of artefacts in the results obtained in this way.

### The SRS itself does not alter plant growth

**Fig. 4:**
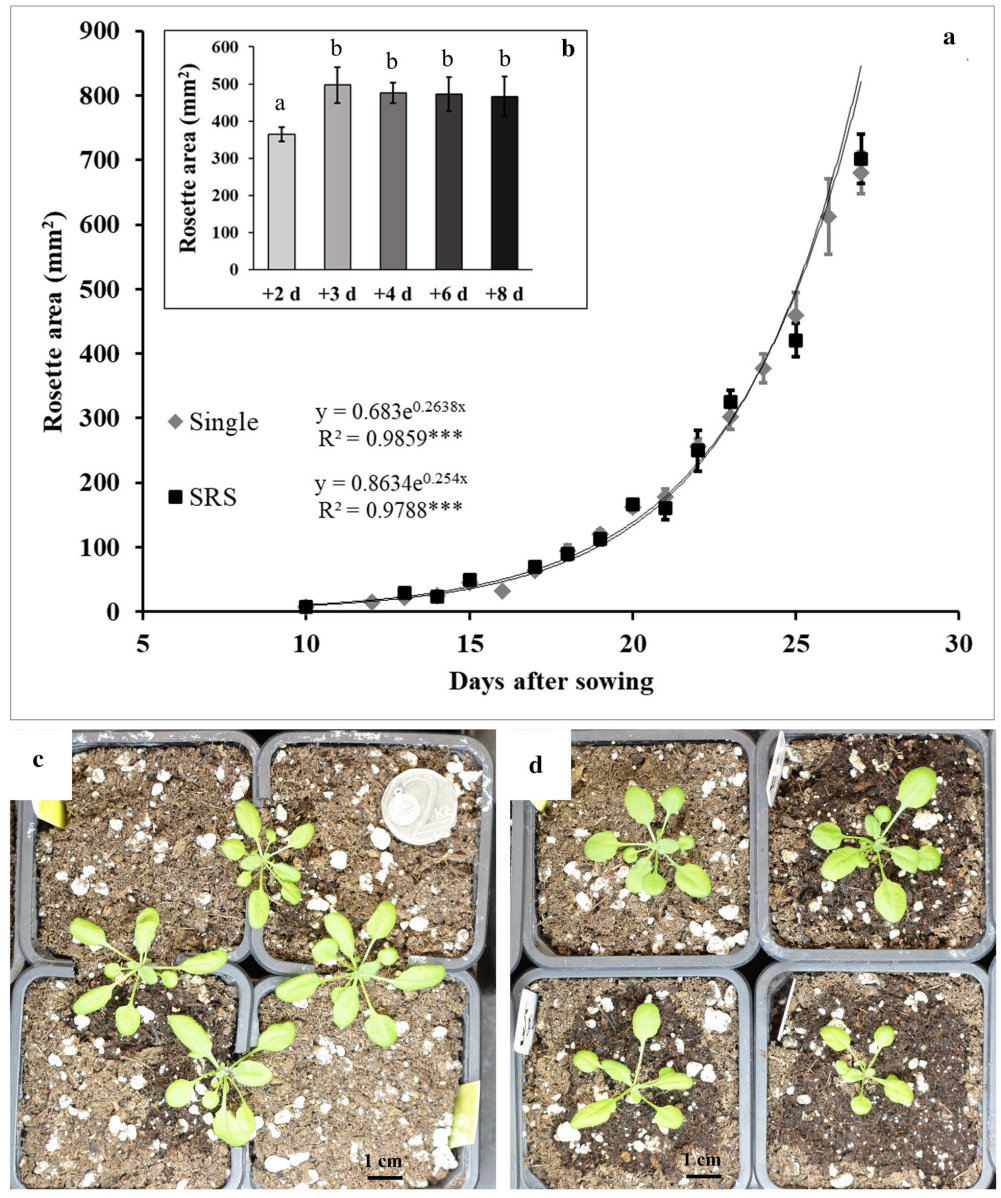
Comparison of the rosette area (mm^2^) of Arabidopsis plants growing either on an SRS or on single pots (**a**). Effect of the time waited between the partial de-rooting, performed 7 days after sowing, and the transplant of plants into the SRS pots on the rosette area of Arabidopsis plants 25 days after sowing (**b**). Treatments with different letters present statistically significant differences for p < 0.05. Visual comparison of Arabidopsis plants growing on an SRS (**c**) and on single pots (**d**). The black bar is equivalent to 1 cm

The comparison between the growth curves of plants placed in an SRS (Fig. 4c) and plants growing on individual pots (Fig. 4d) confirms that the SRS itself does not affect plant development (Fig. 4a). Additionally, the time gap between the de-rooting procedure and the transplant does not seem to be critical for plant development, provided newly formed roots have reached a minimum length (Fig. 4b). This minimum root length will depend on the type of SRS employed, since the distance between the separate growth media that roots need to cover varies among them. In the case of SRS obtained stacking square plastic pots, the minimum root length was around 1.5 cm, which in PDR plants was reached 3 days after the de-rooting procedure, on average. When the transfer to the SRS was done earlier than 3 days after de-rooting, plants were still able to survive the transplant, but their growth rate was reduced, leading to a smaller leaf area (Fig. 4b). It is worth mentioning that longer waiting periods after de-rooting (6–8 days) made the process of plant transfer into the SRS significantly more difficult. This was due to the fact that these plants had longer roots, and as such were more prone to root tangling than the plants transplanted 3 to 4 days after de-rooting. Although these effects did not alter the development of the plants that were successfully transplanted, it did increase both the number of plants lost during transplantation and the time required for its completion.

According to these results, the best strategy to establish a SRS in *Arabidopsis thaliana* plants is the partial de-rooting of the young seedlings, which allows plants to uptake some water and nutrients through the remnants of the root [27], as opposed to the complete excision of the root system. If the goal is to establish the SRS with plants as young as possible, a partial de-rooting 4 DAS is probably the best approach. However, in those situations in which the time of the SRS establishment is not crucial, 7 DAS seems to be the optimal time to perform the partial de-rooting (Table 2). After de-rooting, it is recommended to wait until the newly formed roots are long enough to comfortably reach the different growth media, but not so long that the roots are prone to tangling.

### SRSs can be used to solve problems inherent to certain experimental designs

As mentioned above, SRSs have found many applications in plant sciences; however, this technique may not have reached its full potential yet. SRSs could be used to overcome problems inherent to experimental set-ups in several areas of plant sciences. For example, during experiments involving drought, any time the application of a compound is required it must be done in a water-based solution form, leading to plant rehydration and delay or amelioration of drought effects. SRSs offer a way to overcome this difficulty, since they allow to supply the required compound to one side of the root system, which can then be cut after the compound has been absorbed by the plant, thus limiting the rehydration process. This technique is particularly interesting for studies employing inducible mutant lines, which need to be activated using dexamethasone (DEX), estradiol or another analogue soluble compound. Besides, the same SRS system might be also useful for studying the impact of naturally or synthetically derived compounds such as plant hormones on stress-related responses.

**Fig. 5:**
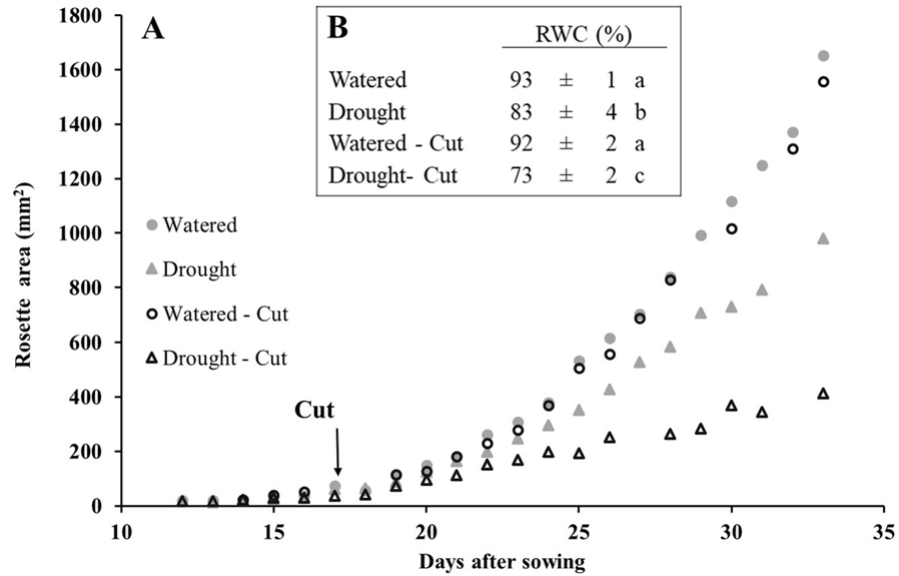
Effect of cutting half of the root system and keeping the remaining half either watered or under drought conditions on rosette area of Arabidopsis plants growing on an SRS (**a**) and on the relative water content (RWC) 34 days after sowing (**b**). Water was withdrawn from drought treatment starting 10 DAS, plants were watered one more time 16 days after sowing, and the watered side of the SRS was cut 17 DAS. Treatments with different letters present statistically significant differences for p < 0.05

To this end, an inducible system was examined in *Arabidopsis thaliana* plants growing in an SRS with soil (Fig. 4c). After a week under either normal watering conditions or water deprivation, 16 DAS one side of the SRS was watered one more time, to simulate the application of the test compound, and the following day the side of the SRS that had been watered was cut. This procedure barely affected the growth of the plants under watered conditions (Fig. 5a), which suggests that plants were able to withstand the effects of excising half of their root system and still grow normally. The rosette presented homogeneous growth, without favoring the leaves of any specific side of the SRS. This falls in line with studies that show that half of the root system is capable of supporting the growth of the entire aerial part [62, 63]. In the case of plants grown under water deprivation shoot growth was also maintained after removal of the watered half of the SRS, since at that point the soil was not completely dry. However, the effect of drought stress on rosette expansion could be observed earlier and in a bigger extent than in uncut drought plants (Fig. 5a), and the relative water content (RWC) was further decreased (Fig. 5b).

A similar experiment was set using the *Hordeum vulgare* cytokinin oxidase/dehydrogenase (*HvCKX*) and isopentenyl transferase overexpressing Arabidopsis transgenic lines *proCaMV35S *> GR > *HvCKX2* (CKX) [64] and *proCaMV35S *> GR > *ipt* (IPT) [65]. These plants, once activated with DEX, display an increased expression of key enzymes of cytokinin catabolism, which has been shown to alter drought tolerance in barley (*Hordeum vulgare*) [66], tomato (*Solanum lycopersicum*) [67] and Arabidopsis [68]. Using these inducible mutants allows the effect of alterations in cytokinin metabolism to be studied at specific stages of the stress response. However, the necessity to apply water in order to activate them can be problematic in the case of drought stress experiments, since it will result in plant rehydration. Here, we discuss the possibility of employing a SRS to overcome this particular limitation of inducible mutants. After growing the plants in a SRS, 10 DAS water was withdrawn, 16 DAS plants were activated with DEX and, 1 day later, the activated side of the root system was cut. Similarly as observed in wild-type plants, in the case of uncut IPT plants no significant differences in rosette area were observed between watered and drought treatments until 26 DAS (Table 3). The delay was even more pronounced in the case of uncut CKX plants, which did not show symptoms of water deprivation until 30 DAS. Growing these plants in an SRS allows the application of the DEX-containing watering solution to only one side of the root system which, once full activation has been achieved (24 h seems to be enough [69]), can be cut off from the plant, therefore stopping the rehydration process. This procedure significantly promotes the aforementioned onset of drought stress (21 and 24 DAS in the case of IPT and CKX plant, respectively-Table 3), allowing the study of the effect of water deprivation at earlier stages of plant development.

**Table 3:** Rosette area (mm^2^) and RWC (%) of *Arabidopsis thaliana* wild type (WT), *proCaMV35S *> GR > *HvCKX2* (CKX) and *proCaMV35S *> GR > *ipt* (IPT) plants after half of the SRS had been activated with DEX and cut from the main plant the following day

	Area 21 DAS		Area 24 DAS		Area 26 DAS		Area 30 DAS		Final RWC
WT									
Watered	183 ± 6 a	10%	372 ± 19 a	19%	612 ± 22 a	28%	1121 ± 40 a	33%	92 ± 2 a
Drought	165 ± 6 a		300 ± 17 b		441 ± 20 b		745 ± 34 b		83 ± 3 b
Watered-Cut	182 ± 10 a	38%	369 ± 22 a	46%	558 ± 36 a	55%	1003 ± 63 a	63%	92 ± 2 a
Dorught-Cut	112 ± 7 b		197 ± 19 c		253 ± 20 c		368 ± 33 c		74 ± 2 c
CKX									
Watered	181 ± 9 a	7%	301 ± 21 a	7%	442 ± 26 a	9%	815 ± 48 a	17%	91 ± 1 a
Drought	168 ± 8 ab		279 ± 21 a		402 ± 22 a		678 ± 34 b		85 ± 2 b
Watered-Cut	151 ± 7 ab	10%	280 ± 18 a	28%	406 ± 26 a	31%	723 ± 49 ab	39%	90 ± 1 a
Dorught-Cut	137 ± 5 b		202 ± 13 b		282 ± 13 b		443 ± 20 c		76 ± 2 c
IPT									
Watered	178 ± 10 a	8%	375 ± 24 a	13%	567 ± 32 a	20%	1025 ± 50 a	26%	88 ± 1 a
Drought	165 ± 11 a		327 ± 26 a		454 ± 26 b		756 ± 42 b		78 ± 2 b
Watered-Cut	177 ± 21 a	22%	362 ± 40 a	32%	527 ± 54 ab	37%	938 ± 71 a	56%	86 ± 1 a
Dorught-Cut	138 ± 7 b		247 ± 17 b		331 ± 18 c		410 ± 24 c		72 ± 2 c

Different letters mean statistically significant differences (within each genotype) for p < 0.05, according to Student’s *t* test (n ≥ 10)

The percentual number indicates the decrease in rosette area (in %) between watered and drought plants and between watered-cut and drought-cut plants. Water was withdrawn from drought treatment starting 10 DAS, plants were watered one more time 16 days after sowing, and the watered side of the SRS was cut 17 DAS. Afterwards, the remaining half of the root system was kept under either watered or drought conditions

**Fig. 6:**
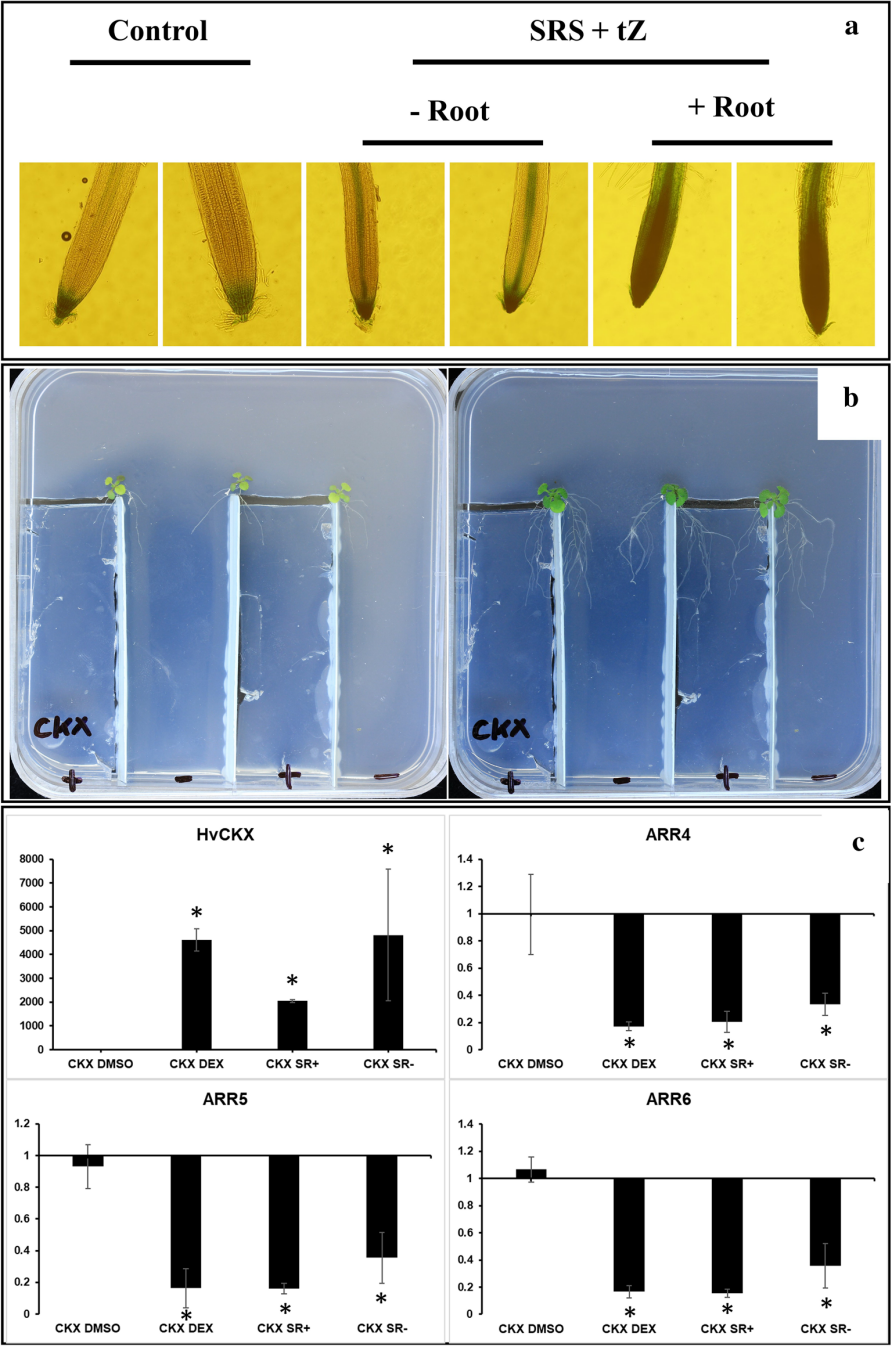
Visualization of the cytokinin signalling in Arabidopsis *ARR5::GUS* transgenic lines (**a**). 10 µM tZ were applied to one side of the SRS (+ Root) and the increase on expression of *ARR5* was observed on the other half (−Root). Picture of Arabidopsis DEX-inducible CKX overexpressing plants transplanted into the custom SRS dishes (**b**) containing patches of MS (**−**) and MS + DEX (**+**) at the moment of transplant (left) and after 5 days (right). RT-qPCR analysis of the relative expression of *HvCKX* and *ARR4*, *5* and *6* on roots of Arabidopsis *proCaMV35S* > GR > *HvCKX2* line (CKX-**c**) after either the whole root system (CKX DEX) or half of the SRS had been activated (CKX SR; + is the activated half and − the non-activated one). Asterisks indicated statistically significant differences respect to non-activated CKX plants (DMSO) for p < 0.05

However, the applicability of this technique is going to be limited by the mobility of each particular compound within the plant. Depending on the nature of the compound and the plant species under study, the applied compounds may be distributed all over the plant, stay in the place of application, or be translocated only to the aerial part, without being distributed to the other side of the SRS [70, 71]. Using *Arabidopsis thaliana* *ARR5::GUS* transgenic line, we tracked the evolution of the cytokinin signalling across the root split after exogenously applying cytokinins to one half of the SRS. The results from GUS staining suggest that the cytokinin signalling cascade can travel from one side of the SRS to the other (Fig. 6a). Similarly, it was necessary to validate that, when using DEX-inducible mutant lines, activation of one half of the root system will result in a comparable expression of the transgene in the opposite half. RT-qPCR analysis performed on Arabidopsis CKX plants growing on a SRS, with only half of the root system being supplemented with DEX (Fig. 6b). These plants displayed the typical phenotype associated with CKX overexpressing plants [69] and the RT-qPCR showed a comparable expression at both sides of the SRS of both the transgene *HvCKX* and key marker genes *ARR4*-*6* of the cytokinin signalling pathway (Fig. 6c), confirming that the application of DEX to one part of the root system is enough to activate the whole plant. Therefore, when designing any experiment that includes SRSs, it is essential to take into account the mobility of the compounds that are going to be applied. Additionally, in research involving low mobility compounds, SRSs allow the study of their temporary effect on plant development, since once the half of the SRS that contains the given compound is cut off, the rest of the plant can recover from its effects.

## Conclusions

**Fig. 7:**
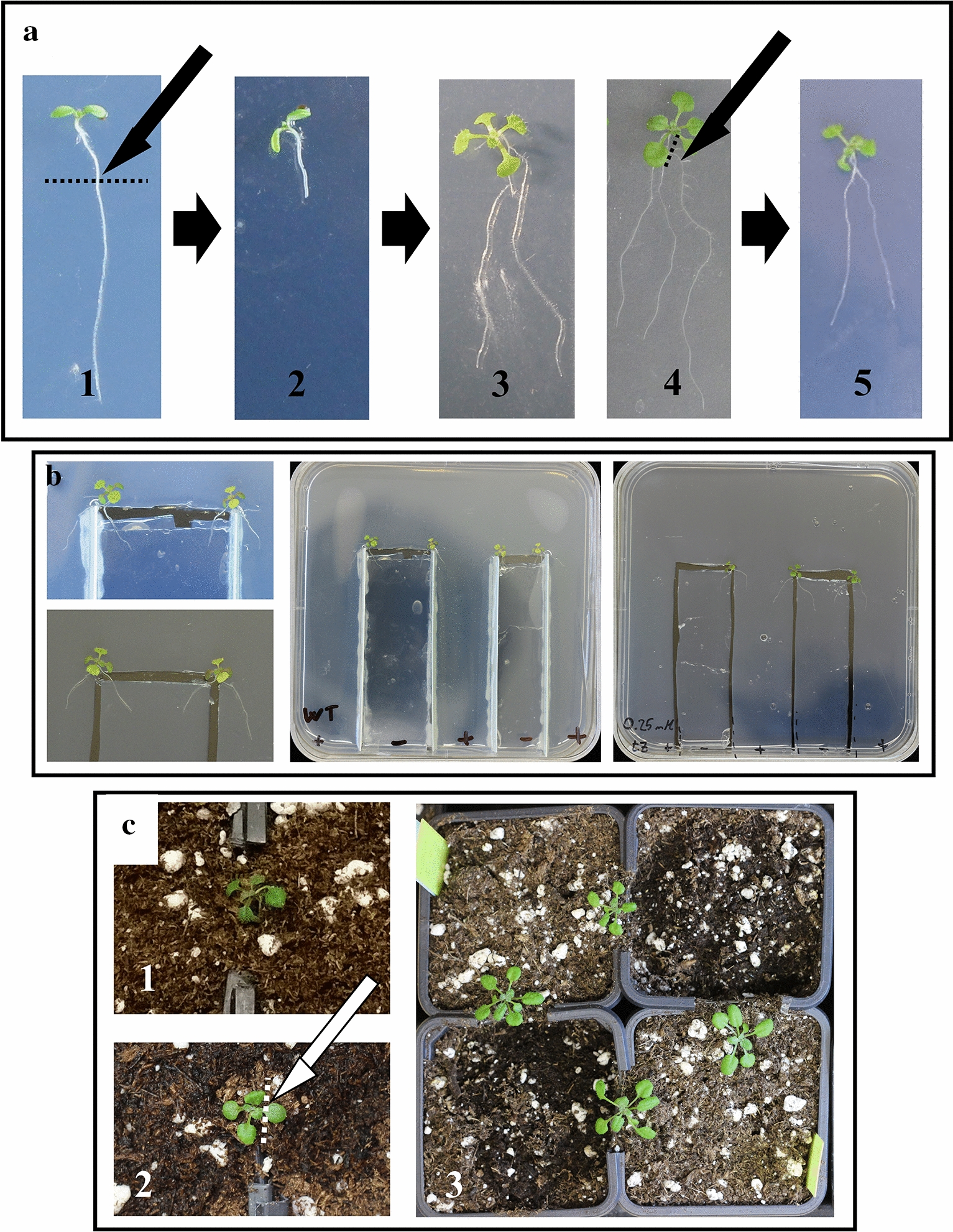
Overview of the method for establishing an SRS in young *Arabidopsis thaliana* seedlings. The de-rooting procedure (**a**) was done by cutting the main root of the seedling with a scalpel (1), leaving around 0.5 cm attached to the hypocotyl (2). The plants were then allowed to re-root, generating plants with either symmetric (3) or asymmetric (4) root systems. The roots of the latter can be trimmed with a scalpel or micro-cutting scissors, thus leaving a symmetric root system (5). Those plants with symmetric root systems (3 and 5) are suitable for transplant into petri dishes (**b**), either with plastic spacers (centre) or without them (left), or into custom pots (**c**). Pot-based SRSs can be used in drought studies, since they allow the application of water-soluble compounds to one side of the root system, which can later be cut (bottom left), leaving the plants growing on the water-deprived soil (right)

SRSs can have many applications in plant sciences, but their implementation can be difficult and time-consuming, depending on the experimental design. Here, we optimized and explained in detail a method for establishing an SRS in young *Arabidopsis thaliana* seedlings, both in vitro and in soil (Fig. 7). This method, which favours partial de-rooting over complete de-rooting of the seedlings, minimized the recovery time, thus allowing the establishment of the SRS on younger plants, while maximizing plant survival. The analysis of the changes in plant leaf proteome following the de-rooting procedure highlighted the distinct metabolic alterations that TDR and PDR plants undergo during the healing process. Even though plants were able to develop normally after the establishment of the SRS, these metabolic alterations should be considered while discussing the results of any experiment that employs SRSs. Lastly, we validated a method for using SRSs in drought experiments, which allows the application of chemical compounds to only one half of the SRS, which can later be cut from the rest of the plant, thus avoiding rehydration and maintaining the drought stress.

## Materials and methods

### Plant material and growth conditions

Seeds of *Arabidopsis thaliana* transgenic lines *proCaMV35S *> GR > *ipt* (IPT) [65] and *proCaMV35S *> GR > *HvCKX2* (CKX) [64], and corresponding wild type (Col-0) were sown on square petri dishes filled with 50 mL of full Murashige and Skoog (MS) medium (pH = 5.75) containing 0.6% (w/v) MES and 1.2% (w/v) agar. After 2 days of stratification at 4 °C, plants were moved to growth chambers with a 16-h day/8-h night light regime (110 µmol photons m^−2^ s^−1^), 21 °C and 19 °C, respectively.

For in-soil experiments seedlings of different developmental stages were transplanted into standardized plastic pots (7 × 7 × 8 cm) filled with Steckmedium substrate (Klasmann-Deilmann, Geeste, Germany) and kept at the same conditions as in vitro plants. Pots were watered three times per week with 20 mL of water.

DEX-inducible and wild-type lines were activated at different developmental stages by transferring plants to full-strength MS medium supplemented with either 10 µM DEX dissolved in 0.1% (v/v) dimethyl sulfoxide (DMSO) or 0.1% (v/v) DMSO (mock). In the case of in-soil experiments, activation was achieved by watering plants with 20 mL of 10 µM DEX dissolved in 0.1% (v/v) DMSO.

### Establishment of the split-root system

#### Cutting of the roots

*Arabidopsis thaliana* plants were grown in the conditions described above until the chosen moment of cutting. In order to optimize the method for the establishment of the SRS, different cutting days were compared, ranging from 4 to 15 DAS. The cut was performed directly on the agar using a sharp sterilized blade, and the excised roots were removed. By not moving the plants out of the dishes it is possible to minimize the level of manipulation and associated stress. The cut was done right at the junction between root and hypocotyl in the case of TDR plants, or around 1 centimetre below the hypocotyl in the case of PDR plants. After the cutting procedure, it is important to make sure that the end of the hypocotyl/root is in contact with the agar in order to prevent dehydration and facilitate the new rooting. The petri dishes were then closed, sealed, and incubated in a growth chamber.

Note: If for whatever reason the cut cannot be performed on the agar (as could be the case with plants grown in hydroponics), it is recommended to place a piece of sterilized and water-dampened filter paper over the cover of a petri dish. This offers a hard surface against which to perform the cut, while keeping the roots from rapidly drying.

#### Preparation of the custom petri dishes and pots

For the preparation of the custom split-root petri dishes, plastic pot label strips were longitudinally cut into narrower, around 1 cm-wide, strips. The strips were glued to square petri dishes, three per dish, thus creating four separate areas. The area separators created this way did not reach the top of the dish, since having a flat area where to place the plants proved to be more convenient than having to place them over the edge of the spacer. The only purpose of these separators is to keep the roots from growing into adjacent areas and, therefore, may not be necessary for short-term experiments. These custom plates can be sterilized with ethanol and UV light before being used (depending on the materials of the dishes and spacers, autoclaving may not be possible).

For the preparation of the heterogeneous media, first we poured the hot liquid control media (either MS or MS + 0.1% DMSO, depending on the experiment), making sure that it covered the whole dish. Once it had cooled down and solidified, the part of the media confined within two of the separate areas was cut out with an sterile scalpel and taken out of the dish, thus leaving an empty space in the media. Afterward, a piece of the same width and slightly smaller height was cut out from an unmodified petri dish that had been filled with MS media supplemented with whatever treatment was relevant for the experiment. This piece was then inserted into the empty gap of the modified petri dish, leaving a small (a couple of mms) gap between both media, in order to avoid any diffusion between them.

The custom split-root pots were prepared using standardized rectangular plastic pots (7 × 7 × 8 cm). A small piece (2.5 cm wide, 0.5 cm deep) was cut from the top-centre part of two adjacent sides of the pot. Four pots were arranged in a square, with their respective cut parts placed against each other, and kept in place using wide tape. A piece of tape was also used to close the gap between each pair of cut parts of the pots. Lastly, the pots were filled with Steckmedium (Klasmann-Deilmann) substrate up to slightly above the cut, creating a thin bridge of soil where the plants will be placed during the establishment of the SRS. Right before the transplant, two shallow cavities were carved at both sides of the gap between each pair of pots, being careful to leave enough soil to keep the bridge between pots covered. These cavities will allow the placing of the roots with their tips already facing downwards, instead of lying flat against the surface of the soil.

Note: By filling the pots up above the cut, each pair of adjacent pot is effectively linked by a small bridge of soil. During the experiments, watering of each pot was performed very carefully, and no horizontal movement of watering solution was observed across this bridge. Alternatively, it is possible to fill the pots just up to the edge of the cut, therefore avoiding the formation of the aforementioned bridge of soil. However, this offers a much less stable area to place the plant during transplantation.

#### Transplanting into the petri dishes and pots

De-rooted plants were left to re-root until the new roots were long enough for their transplant into either the custom petri dishes or the custom pots described above. The minimal length for a successful transplant was around 1.5 cm. Only those plants that presented symmetrical root systems (i.e. that had the same number and length of roots at both sides of the split) were chosen for transplantation. The tip of a tweezer was placed below one of the leaves, without closing it, and the plant was lifted, while another tweezer was used to keep both sides of the root system separated by gently pushing one away from the other. Afterwards, the plant was moved over the custom petri dish and slowly lowered, making sure that the root that is not being pushed away with the tweezer touched the media first. This prevents the root from hanging freely and makes the remaining of the transplant easier. Then, the shoot was lowered onto the media and, lastly, the other root was lowered and placed at the side of the plastic spacer opposite to the first root. Lastly, the petri dishes were re-sealed and placed in the growth chamber. It is recommended to perform this transplant under sterile conditions, under a flow box, even though for very short experiments contamination may not be an issue. In the case of plants transplanted into the custom pots, the procedure was performed in a very similar way; except that it was not done under sterile conditions, since the soil was not sterile itself. First, one root was placed into the cavity carved in the soil at one side of the bridge, then the shoot was lowered onto the soil bridge connecting the two pots and, lastly, the second root was placed in the cavity of the opposite pot. Both cavities were then carefully filled with soil, making sure that the roots were completely covered in order to avoid desiccation. Afterwards, the pots were watered to facilitate the taking of the roots.

### Phenotypic analysis

Plant images were taken at different stages of development, both pre- and post- cut and pre- and post-transplant, using a Canon EOS 600D (Canon Inc., Tokyo, Japan) digital camera (n ≥ 10). Rosette area was then estimated using Image-J (https://imagej.nih.gov/ij/).

The relative growth rate (RGR) was calculated as follows (n ≥ 10):

$${\text{RGR }} = \, \left( {{\text{ln area}}_{\text{t1}} {-}{\text{ ln area}}_{{{\text{t}}0}} } \right)/\left( {{\text{t1 }}{-}{\text{ t}}0} \right)$$

where area_t1_ is the rosette area at a given measurement time, area_t0_ is the rosette area at the immediately preceding measurement time, and t1 – t0 is the number of days between those two time points. The RGR was then used to calculate the plant recovery time, which is the number of days passed between the de-rooting procedure and the moment in which the RGR was no longer decreased in comparison to uncut plants.

The relative water content (RWC) was calculated using one leaf per plant (n ≥ 7) as follows:

$${\text{RWC }} = { 1}00 \, * \, \left( {{\text{FW }} - {\text{ DW}}} \right)/\left( {{\text{TW }} - {\text{ DW}}} \right)$$

where FW is the fresh weight of the leaf, TW is the turgid weight, measured after keeping the leaf in water for 24 h at 4 °C, and DW is the dry weight, measured after 24 h in an oven at 80 °C.

### *ARR5::GUS* assay

An SRS was established using *Arabidopsis thaliana* *ARR5::GUS* transgenic plants, following the method described above. The plants were transferred to custom petri dishes containing separate patches of MS and MS + 10 µM transzeatin (tZ) in 0.1% DMSO. One side of the root system of each plant was placed on the medium supplemented with tZ and the other on the normal MS medium. Plants were kept for 2 days in these heterogenous growth conditions before performing the GUS staining.

The staining was performed by submerging the plants in a staining solution containing 0.1 M phosphate buffer (pH = 7.2), 0.5 mM K_3_[Fe(CN)_6_], 1% TRITON-X114, and 0.1 mg/mL X-GlcA in DMSO; and applying vacuum for 10 min. Then, the pressure was slowly restored, and plants were incubated overnight in the dark. Afterwards, the plants were washed with a destaining solution consisting of 70% (v/v) ethanol and 20% (v/v) chloroform. Pictures were taken using an Olympus IX70 microscope.

### Quantitative RT-PCR

13-day old Arabidopsis *proCaMV35S *> GR > *HvCKX2* plants were transferred to a customised petri dish in order to establish an SRS (Fig. 6b). One side of the root was placed on an isolated patch of agar containing 10 µM DEX in 0.04% v/v DMSO, with the other side being placed on agar containing 0.04% v/v DMSO. Control plants were placed in a homogeneous medium containing either 10 µM DEX in 0.04% v/v DMSO (positive controls) or 0.04% v/v DMSO (negative controls). 7 days after activation, the roots of the plants were harvested (the activated and inactivated halves of SR plants being harvested separately), roots from the same petri dish were pooled together, and the resulting samples immediately frozen in liquid nitrogen (n = 3). The total RNA was extracted according to a combined protocol of TRI Reagent RT (Molecular Research Center, Cincinnati, Ohio, United States) and the RNeasy Kit (Qiagen, Hilden, Germany) with on-column DNase (Qiagen) digestion. All individual reactions were done in triplicate on a LightCycler 480 (Roche Diagnostics, Risch-Rotkreuz, Switzerland) using the UPL system (Roche Applied Science, Penzberg, Germany), as described by Skalák et al. [69], with *UBQ10* and *ACT2* as reference genes. The list of primers can be found in Additional file 1: Table S5.

### Proteomic analysis

For the proteomic analysis, *Arabidopsis thaliana* wild type (Col-0) plants were grown in vitro for 7 days in the same wroth conditions as described above, before being totally or partially de-rooted. Leaf samples were taken at different stages of post-derooting development, which corresponded with the moment shortly after the cut (4 h after de-rooting; early response), the time at which the difference in the RGR of TDR plants was starting to recover (5 days after de-rooting; recovery), and the time at which the RGR of TDR plants had reached that of uncut plants (10 days after de-rooting; acclimation). Each sample consisted of the cotyledons, first two true leaves, or 3rd and 4th leaves, in the case of 4 h, 5 days, and 10 days after de-rooting, respectively, of all the plants growing in at least two petri dishes (8 plants per dish) pooled together and ground in a mortar with liquid nitrogen. The harvested leaves were fully developed and in a similar developmental stage across treatments. Two independent replicas per treatment and time point were used for the whole proteome profiling, and a third replicate was used for targeted validations. Approximately 0.1 g of FW was extracted following the combined metabolite/protein extraction method described by Salem et al. [72]. The polar phase was then used for semi-quantitative metabolomic profiling, as described by Saiz-Fernández et al. [73]. The pellets were solubilized and digested with immobilized trypsin (Promega, Madison, Wisconsin, United States) overnight, and desalted by C18 SPE, following the method described by Cerna et al. [74]. Semi-quantitative proteomic analyses were performed using a gel-free shotgun protocol based on nano-HPLC and MS/MS [74]. Briefly, tryptic digests were dissolved in 0.1% (v/v) formic acid in 4% (v/v) acetonitrile, and then aliquots corresponding to 5 μg of peptide were analyzed by nanoflow C18 reverse-phase liquid chromatography using a 15 cm column (0.1 mm inner diameter; Zorbax, Agilent, Santa Clara, California, United States) and a Dionex Ultimate 3000 RSLC nano-UPLC system (Thermo, Bremen, Germany) directly coupled to a CaptiveSpray nanoESI source (Bruker, Bremen, Germany) and a UHR maXis impact q-TOF mass spectrometer (Bruker). Peptides were eluted for up to a 120 min with a 4–40% acetonitrile gradient. Spectra were acquired at 2 Hz (MS) and 10–20 Hz (MS/MS) using an intensity-dependent mode with a total cycle time of 7 s. The measured spectra were extracted and processed as described previously by Dufková et al. [75]. In brief, recalibrated MGF mascot generic format files were searched against the Araport Arabidopsis protein database by Proteome Discoverer 2.1, employing Sequest HT, MS Amanda, and Mascot 2.4 with the following parameters: enzyme—trypsin, max two missed cleavage sites; mass tolerance—35 ppm (MS) and 0.1 Da (MS/MS); modifications—up to three dynamic modifications including Met oxidation, Asn/Gln deamidation, Lys methylation, N-terminal Gln/Glu to pyro-Glu, N-terminal acetylation, and N-terminal Met loss. The quantitative differences were evaluated by calculating the normalized number of peptide spectral matches. Only differentially abundant proteins with at least one detectable proteotypic peptide, available quantitative data in both biological replicates, and absolute fold-changes respect to controls greater than 2 were selected for a targeted validation in a third biological replicate via SRM-based analyses (Skyline 3.1, MacCossLab Software, https://skyline.gs.washington.edu) that employed an LC interface connected to TSQ Quantiva (Thermo) (at least three most intensive y-ions; Q1 and Q3 resolution 0.4 Da; cycle time < 4 s). Statistical significance compared to control was validated by a *t* test (p < 0.05). Data are presented as the ratio between the protein abundance found in de-rooted plants (either PDR or TDR) and uncut plants, at each time point.

## Supplementary Information

**Additional file 1: Tables S1–S4.** Spreadsheet showing the Log10 fold changes of the proteins related to different biological processes. **Table S5.** The list of primers used for RT-qPCR.

## Data Availability

The datasets used and/or analysed during the current study are available from the corresponding author on reasonable request.

## Abbreviations

IPT: *proCaMV35S *> GR > *ipt*
CKX: *proCaMV35S *> GR > *HvCKX2*
DAS: Days after sowing
DEX: Dexamethasone
DW: Dry weight
FW: Fresh weight
HSP: Heat-shock protein
*HvCKX*: *Hordeum vulgare* cytokinin oxidase/dehydrogenase
PDR: Partial de-rooting
RWC: Relative water content
RGR: Relative growth rate
SRS: Split-root system
TDR: Total de-rooting
